# Efficacy of Combination Therapy with the JAK Inhibitor Baricitinib in the Treatment of COVID-19

**DOI:** 10.1007/s42399-022-01121-4

**Published:** 2022-01-21

**Authors:** Brendan L. Thoms, Jeanne Gosselin, Bonita Libman, Benjamin Littenberg, Ralph C. Budd

**Affiliations:** 1 Division of Rheumatology and Clinical Immunology, Department of Medicine, University of Vermont Medical Center, 111 Colchester Avenue, Burlington, VT 05401 USA; 2 Rheumatology and Clinical Immunology Division, Department of Medicine, The Larner College of Medicine at the University of Vermont, Burlington, VT 05405 USA; 3 Division of General Internal Medicine Research, Department of Medicine, The University of Vermont Larner College of Medicine, Burlington, VT 05405 USA; 4 Vermont Center for Immunology and Infectious Diseases, The University of Vermont Larner College of Medicine, Burlington, VT 05405 USA

**Keywords:** Coronavirus disease-19 (COVID-19), JAK inhibitor, Baricitinib, Cytokine storm, Critical care

## Abstract

Coronavirus disease-19 (COVID-19), resulting from infection with SARS-CoV-2, spans a wide spectrum of illness. In severely ill patients, highly elevated serum levels of certain cytokines and considerable cytolytic T cell infiltrates in the lungs have been observed. These same patients may bear low to negligible viral burdens suggesting that an overactive immune response, often termed cytokine storm, contributes to the severity of COVID-19. We report the safety and efficacy of baricitinib combined with remdesivir and dexamethasone in a retrospective review of 45 hospitalized patients with COVID-19 pneumonia at a tertiary academic medical center. Patients received 7-day course of baricitinib, 5-day course of remdesivir, and 10-day course of dexamethasone. Clinical status and biomarkers were obtained daily. Outcomes assessed include mortality, duration of hospitalization, presence of shock, need for supplemental oxygen, need for non-invasive ventilation, need for mechanical ventilation, and development of thrombosis. Obesity and multiple medical comorbidities were associated with hospitalization in the setting of COVID-19. Treated patients demonstrated rapid declines of C-reactive protein (CRP), ferritin and D-dimer with gradual improvement in hemoglobin, platelet counts, and clinical status. Only 2 of 45 (4.4%) treated patients required mechanical ventilation after initiating treatment, and there were six deaths (13.3%). Only 2 of 45 (4.4%) treated patients required mechanical ventilation after initiating treatment. There were six deaths (13.3%) and these were associated with lower BMI. These findings support the utility of immunosuppression via JAK inhibition in moderate to severe COVID-19 pneumonia.

**Supplementary Information:**

The online version contains supplementary material available at 10.1007/s42399-022-01121-4.

## Background

Severe acute respiratory syndrome coronavirus 2 (SARS-CoV-2) has infected over 272 million people worldwide, resulting in over 5.3 million deaths to date. Dexamethasone use is associated with reduced mortality in hospitalized coronavirus disease 2019 (COVID-19) patients, and similarly, the use of remdesivir is also associated with shortened hospital admissions, improved clinical status, and lower mortality [1, 2]. Despite awareness of these agents, substantial morbidity and mortality due to COVID-19 remain. Studies of various viral infections in animal models have suggested that an excessive immune response can promote hyperinflammation and multi-organ immune-mediated pathology [3]. This is consistent with findings of elevated levels of several cytokines in severe cases of other coronavirus infections including severe acute respiratory syndrome coronavirus 1 (SARS-CoV-1) and Middle East respiratory syndrome coronavirus (MERS-CoV) [4, 5], as well as murine models of the 1918 influenza [6].

SARS-CoV-2 suppresses the initial type I interferon (IFN-I) response that is critical for early control of viral infections [3, 7]. This allows the virus to escape early immune suppression and to replicate more extensively. When the adaptive immune response is later engaged, profound activation of viral-specific cytolytic T cells occurs in response to the high viral burden [8]. This massive expansion of cytolytic T cells causes considerable tissue damage to virally infected cells and potential damage to other uninfected tissues as innocent bystanders [9–11]. This is associated with an elevation in several serum cytokines, such as interferon (IFN)-γ, interleukin (IL)-2, IL-6, IL-10, and the α-chain of the IL-2 receptor (CD25) [12, 13], a phenomenon termed cytokine release syndrome [14]. A multi-center, retrospective study of 150 patients with severe COVID-19 showed a strong association between elevated ferritin and IL-6 levels and adverse clinical outcomes [15]. These observations have collectively suggested that immunosuppressive therapy might mitigate the severity of COVID-19 infections by reducing cytokine release syndrome as well as inhibiting an overactive cytolytic T cell response.

Various anti-cytokine therapies have been considered for the cytokine release syndrome observed in COVID-19. These include tocilizumab, an IL-6 receptor(R) inhibitor [8, 16]; anakinra, an IL-1R antagonist [17]; and inhibitors of the Janus kinase (JAK) pathways [18–22]. Baricitinib, an inhibitor of JAK1 and 2, was approved for treatment of rheumatoid arthritis in 2018 [23, 24]. It inhibits the intracellular cytokine signaling pathways known to be active in severe COVID-19 and reduces levels of IL-2, IL-6, IL-10, IFN-γ, and granulocyte–macrophage colony-stimulating factor (GM-CSF), some of which are important to the cytolytic T cell response. In February 2020, baricitinib was proposed as a potential treatment for COVID-19 based on artificial intelligence algorithms [25]. The authors hypothesized that baricitinib could directly mitigate the inflammatory response triggered by SARS-CoV-2 infection and was also identified baricitinib as a numb-associated kinase (NAK) inhibitor with high-affinity for AP2-associated protein kinase 1 (AAK1). AAK1 was previously described as a crucial regulator of clathrin-mediated endocytosis of coronaviruses and other viruses [26]. This suggested baricitinib could both both directly mitigate the inflammatory response triggered by SARS CoV-2 and have a direct antiviral effects by preventing virus entry into target cells. This additional mechanism could be complementary to the potential benefits of inhibiting the cytokine storm associated with severe COVID-19.

In three case series of patients with COVID-19, baricitinib use was associated with improved oxygenation and reduction of certain inflammatory markers [19–22]. The placebo-controlled Adaptive Covid-19 Treatment Trial 2 (ACTT-2) study found that baricitinib for 14 days plus remdesivir improved recovery times by 1 day and reduced 28-day mortality from 7.8 to 5.1% [19]. More recent work by Guimarães and colleagues reported that a 14-day course of the JAK1 and 3 inhibitor tofacitinib was associated with a nearly 50% lower risk of 28-day mortality (2.8% vs 5.5%) and respiratory failure (18.1% vs 29%) compared with placebo [18]. We report our assessment of the safety and efficacy of approximately 7 days of baricitinib following initiation of treatment with combined remdesivir and dexamethasone in 45 inpatients with moderate to severe COVID-19 pneumonia at a tertiary academic medical center.

## Methods

### Patients

Between July 10, 2020, and February 8, 2021, COVID-19-positive inpatients were considered for treatment with baricitinib 4 mg daily (if glomerular filtration rate (GFR) > 60 mL/min), 2 mg daily (if GFR 30–59 mL/min), or 1 mg daily (if GFR 15–29 mL/min) for up to 7 days or hospital discharge, whichever was shorter. Inclusion criteria included a positive SARS-CoV-2 polymerase chain reaction (PCR) test and one or more of the following: a chest X-ray demonstrating infiltrates compatible with COVID-19 pneumonia, oxygen saturation < 94% on room air, or the need for supplemental oxygen and/or mechanical ventilation. Exclusion criteria included GFR < 15 mL/min or receiving dialysis, active infection with tuberculosis, absolute lymphocyte count < 200 cells/mL, absolute neutrophil count < 500 cells/mL, and suspected drug-induced liver injury. Patients meeting inclusion criteria also received remdesivir 200 mg intravenous (IV) for the first dose followed by 100 mg IV daily for up to 4 days or hospital discharge, whichever came first. Patients also received dexamethasone 6 mg IV daily for 10 days or until hospital discharge, whichever was shorter. Forty-five patients met the criteria and were treated with baricitinib.

### Variables

Daily laboratory results were recorded from routine patient care including hemoglobin (g/dL), white blood cell count (K/cm^2^), lymphocyte count (K/cm^2^), platelet count (K/cm^2^), C-reactive protein (CRP) (g/dL), ferritin (ng/ml), D-dimer (ng/ml), creatinine (g/dL), alanine transaminase (ALT) (U/L), and aspartate transaminase (AST) (U/L). Clinical illness severity was recorded at the time of admission and followed daily using both the National Early Warning Score-2 (NEWS-2 score) and an 8-level ordinal scale of clinical status recommended by the World Health Organization Research and Development Blueprint Group (WHO Clinical Status Score) [27].

### Outcomes

Duration of hospitalization, presence of shock, and development of thrombosis during hospitalization were recorded. The presence and duration of supplemental oxygen requirement, non-invasive ventilation, and mechanical ventilation were followed. Patient discharge destination and mortality were also recorded.

### Statistics

The start date of baricitinib therapy for each patient was arbitrarily set as day 0. All observations were divided into early and late periods. The early treatment period ran from the date of the first available laboratory result (generally day -3) through the day of the first baricitinib dose (Day 0). The late period ran from the day after starting baricitinib until the last available laboratory result. All statistical analyses were performed in Stata 16.1 (StataCorp, LLC, College Station, Texas, USA).

## Results

### Patient Demographics

Of 45 COVID-19 inpatients treated with baricitinib plus dexamethasone and remdesivir, the mean age was 69.5 years (SD ± 16.7) and 53.3% were female. The average patient was obese (BMI 31), and 84.4% of patients had two or more co-existing medical conditions. The most common medical conditions were hypertension (19%), type 2 diabetes mellitus (15%), hyperlipidemia (11%), autoimmune disease (8%), and obstructive airway disease (8%) (Table 1).

**Table 1:** Baseline patient demographics and characteristics of COVID-19-positive inpatients treated with baricitinib/remdesivir/dexamethasone

Characteristic		All Patients (*n* = 45)	
Age—years	Mean age	69.5	± 16.7
Gender—no	Male	21	(46.7)
	Female	24	(53.3)
Ethnicity – no. (%)	Not Hispanic, Latino/a, or Spanish origin	44	(97.8)
	Hispanic or Latino/a	1	(2.2)
Race – no. (%)	White	36	(80.0)
	Asian	4	(8.9)
	African American or Black	2	(4.4)
	Multiracial	1	(2.2)
	Declined	2	(4.4)
Past medical history—no. (%)	Co-existing conditions: None	1	(2.2)
	Co-existing conditions: One	6	(13.3)
	Co-existing conditions: Two or more	38	(84.4)
	Average body mass index	31	
	Body mass index ≥ 30 – no. (%)	22	(48.9)
	Body mass index ≥ 40 – no. (%)	5	(11.1)
	Hypertension	19	(42.2)
	Type 2 diabetes mellitus	15	(33.3)
	Hyperlipidemia	11	(24.4)
	Autoimmune disease (rheumatoid arthritis, psoriatic arthritis, Sjogren's Syndrome, Raynaud's phenomena, giant cell arteritis, ulcerative colitis or pemphigus vulgaris)	8	(17.8)
	Obstructive airway disease (asthma, chronic obstructive pulmonary disease, bronchiectasis or reactive airway disease)	8	(17.8)
	Obstructive sleep apnea	7	(15.6)
	Coronary artery disease	6	(13.3)
	Atrial fibrillation	6	(13.3)
	Heart failure (including: HFpEF, HFrEF or unspecified)	4	(8.9)
	Chronic kidney disease	4	(8.9)
	History of malignancy	3	(6.7)
	Aortic stenosis	3	(6.7)
	Osteoarthritis	3	(6.7)
	Pulmonary hypertension	2	(4.4)
Average duration of symptoms prior to presentation – days		6	
Average severity of illness at presentation	NEWS2 score	5	
	WHO Clinical Status Score	4	
Dose of Baricitinib—no. (%)	Baricitinib 4 mg daily or 2 mg twice daily	34	(75.6)
	Less than baricitinib 4 mg daily or 2 mg twice daily	11	(24.4)
Duration of Baricitinib—no. (%)	Less than 7 days – no	22	(48.9)
	7 days – no	20	(44.4)
	More than 7 days – no	3	(6.7)
Average duration of baricitinib—(days)		6.0	

Plus–minus( ±) values are standard deviation. Patient race and ethnicity are self-reported by patient. Body mass index is calculated by weight (kilograms) divided by square of height (meters). Percentages may not total 100 because of rounding

The mean duration of baricitinib treatment was 6 days, and 44% of patients completed a 7-day course of baricitinib (Table 1). Twenty-two patients did not complete the full 7-day course of baricitinib: 9 patients were discharged before completing the treatment course, medication was discontinued in 8 inpatients in the setting of patient stabilization, 6 patients died, and 1 patient left against medical advice (Table 1). Three patients completed longer courses of baricitinib based on clinical judgment: one patient completed an 11-day course, one patient completed 12 days, and one patient completed two 7-day courses separated by 2 days for a total of 14 days. Of the 45 total patients, 93.3% started baricitinib within 24 h of receiving dexamethasone and remdesivir.

At the time of initial presentation, patients had symptoms for an average of 6 days and had a mean aggregate NEWS-2 score of 5 (medium clinical risk requiring an urgent response). Seventy-five percent of patients had bilateral pneumonia on initial chest X-ray. On admission, 60% of patients required supplemental oxygen, 22% required non-invasive ventilation, and 4% required invasive mechanical ventilation (Table 2).

**Table 2:** Outcomes among of COVID-19 positive inpatients treated with baricitini /remdesivir/dexamethasone. Percentages may not total 100 because of rounding

	All patients (*n* = 45)	
Primary outcomes:		
Mortality over first 7 days following admission – no. (%)	2	(4.4)
Mortality over entire hospitalization – no. (%)	6	(13.3)
Secondary outcomes:		
Diagnosis of hemodynamic shock during hospitalization – no. (%)	4	(8.9)
Thrombosis during hospitalization – no. (%)	-	
Need for vasopressor support during hospitalization – no. (%)	4	(8.9)
Average duration of vasopressor support during hospitalization – days	1.5	
New oxygen requirement during hospitalization – no. (%)	45	(100.0)
Average duration of new oxygen requirement (days)	7.9	
Need for non-invasive ventilation – no. (%)	19	(42.2)
Average duration of non-invasive ventilation (days)	5.1	
Need for mechanical ventilation during hospitalization – no. (%)	4	(8.9)
Average duration of mechanical ventilation during hospitalization (days)	3.75	
Duration of hospitalization (days)	11	
Estimated duration of illness (days)	15	
Discharge status:		
Home or self-care – no. (%)	26	(57.8)
Home with home health services – no. (%)	5	(11.1)
Subacute rehab – no. (%)	4	(8.9)
Skilled nursing facility – no. (%)	2	(4.4)
Transfer to another facility – no. (%)	2	(4.4)
Deceased – no. (%)	6	(13.3)

### Laboratory and Clinical Variables

**Fig. 1:**
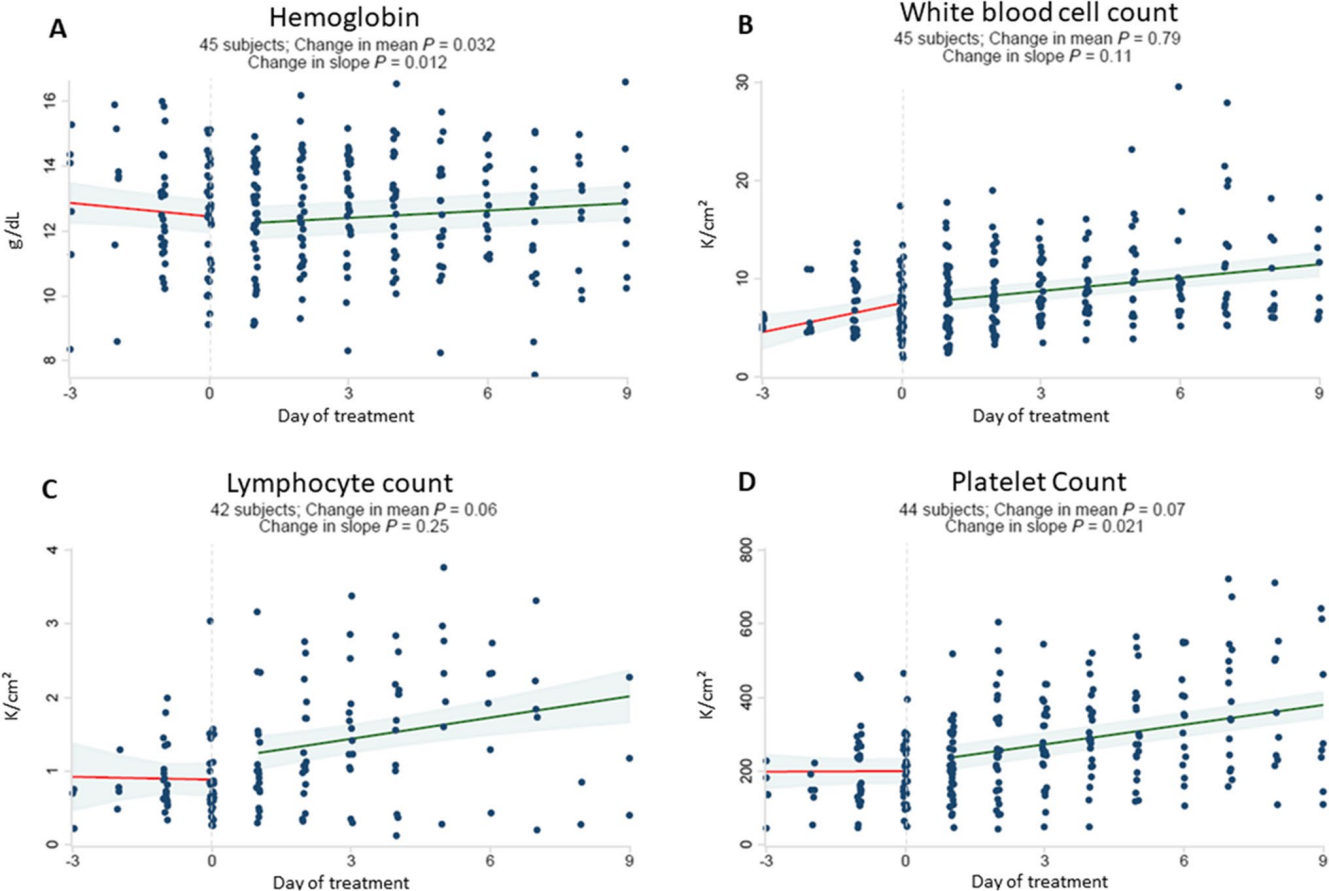
Baricitinib plus dexamethasone and remdesivir therapy reverses downward trajectory of hemoglobin and increases platelet count. Daily laboratory results were recorded from routine patient care including hemoglobin (g/dL) [A], white blood cell count (K/cm^2^) [B], lymphocyte count (K/cm^2^) [C] and platelet count (K/cm^2^) [D]. The start date of baricitinib, dexamethasone and remdesivir therapy for each patient was arbitrarily set as day 0. All observations were divided into early (up to day -3) and late periods (up to day + 9). Linear regressions were perform for each variable and a difference in slope tested between before treatment (day -3 to day 0) vs. after treatment (day + 1 to day + 9)

**Fig. 2:**
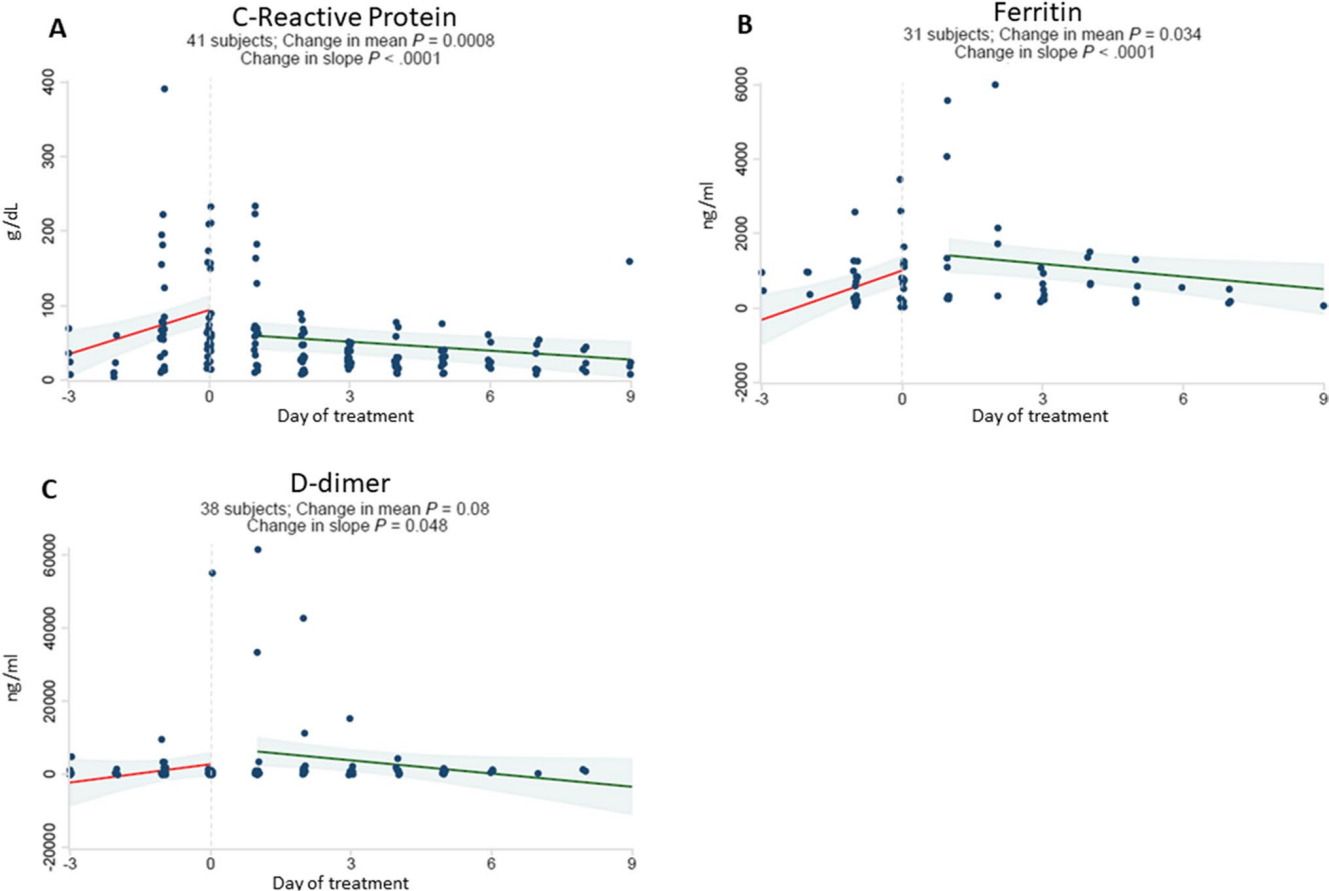
Baricitinib plus dexamethasone and remdesivir therapy reverses upward trajectory of C-reactive protein, ferritin and D-dimer. Daily laboratory results were recorded from routine patient care including CRP (g/dL) [A], ferritin (ng/ml) [B] and D-dimer (ng/ml) [C]. The start date of baricitinib, dexamethasone and remdesivir therapy for each patient was arbitrarily set as day 0. All observations were divided into early (up to day -3) and late periods (up to day + 9). Linear regressions were perform for each variable and a difference in slope tested between before treatment (day -3 to day 0) vs. after treatment (day + 1 to day + 9)

In response to starting baricitinib, dexamethasone, and remdesivir therapy, hemoglobin reversed its downward trend and increased (Fig. 1a). Platelet counts significantly increased, and there was no significant change in white blood cell count (Fig. 1b). Rising levels of CRP, D-dimer, and ferritin also reversed rapidly and significantly after starting baricitinib (Fig. 2). There was no significant change in creatinine or ALT, whereas AST declined significantly following therapy (Supplemental Fig. 1).

**Fig. 3:**
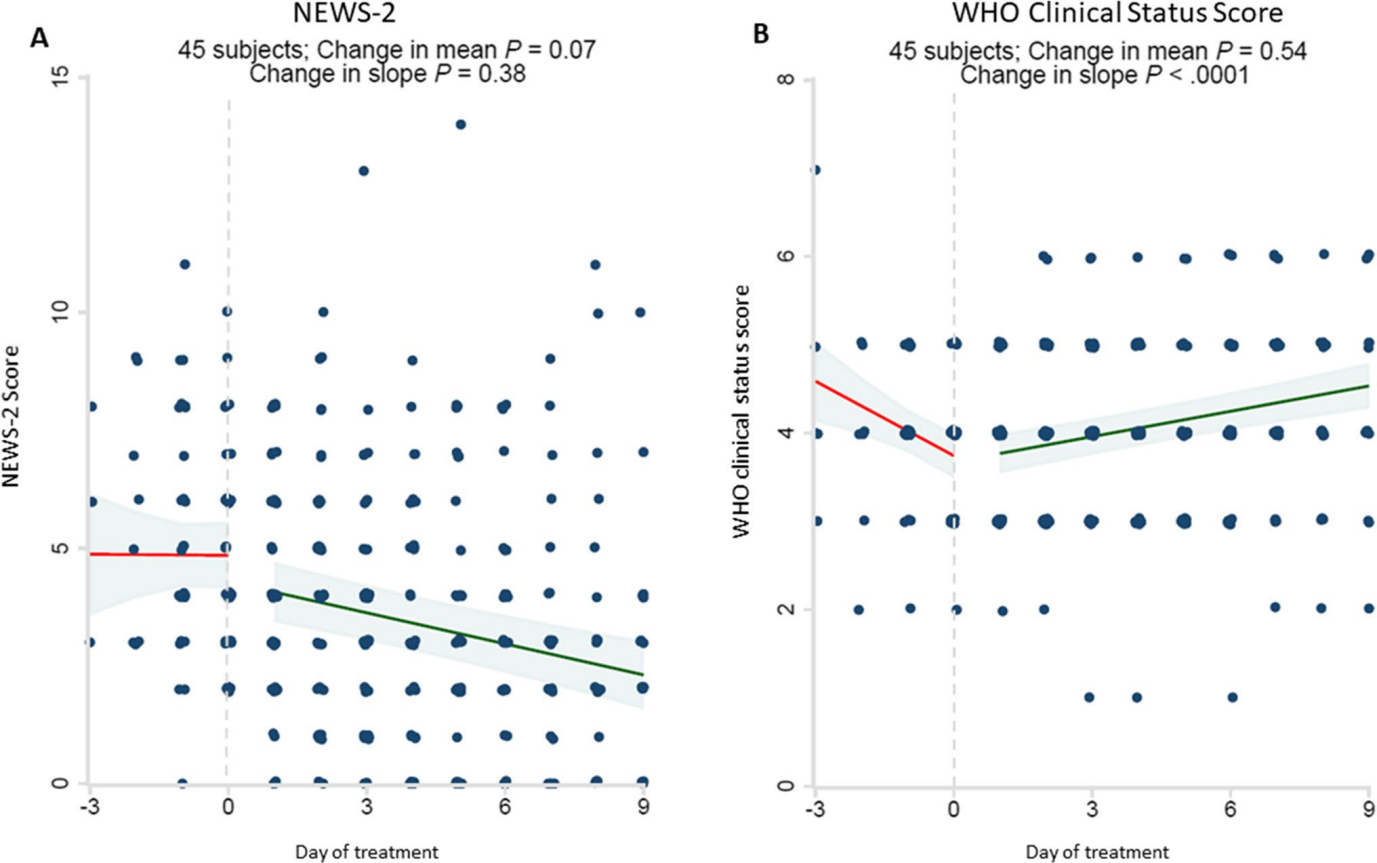
Significant improvement in WHO Clinical Status Score in response to baricitinib plus dexamethasone and remdesivir therapy. Clinical illness severity was recorded at the time of admission and followed daily using the National Early Warning Score-2 (NEWS-2 score) [A] and 8-level ordinal scale of clinical status recommended by the World Health Organization Research and Development Blueprint Group (WHO Clinical Status Score) [B]. There was no statistically significant change in NEWS-2 scores. The start date of baricitinib, dexamethasone and remdesivir therapy for each patient was arbitrarily set as day 0. All observations were divided into early (up to day -3) and late periods (up to day + 9). Linear regressions were performed for each variable and a difference in slope tested between before treatment (day -3 to day 0) vs. after treatment (day + 1 to day + 9)

There was a significant improvement in WHO Clinical Status Score in response to treatment (Fig. 3). NEWS-2 scores also improved with treatment, although this did not achieve statistical significance.

### Patient Outcomes

The average duration of hospitalization was 11 days. Four patients (8.9%) experienced hemodynamic shock. All patients required supplemental oxygen at some point during their admission, and 19 required non-invasive ventilation (42.2%) with an average duration of 5 days. Four patients required mechanical ventilation (8.9%) during hospitalization, with an average duration of 3.75 days. Only two patients required mechanical ventilation following initiation of baricitinib (one patient was transferred from an outside hospital and a second patient was intubated at time of initial presentation prior to starting baricitinib). Of the total study patients, 68.9% were discharged home with self-care or home health services, 13.3% were discharged to subacute rehabilitation or a skilled nursing facility, and 4.4% were transferred to another facility. There were 6 deaths (13.3%) (Table 2).

### Subgroup Analysis

Of the 6 treated patients who died, 2 died within the first 7 days of hospitalization. Deaths occurred in patients who were significantly older (mean age 84.5 [SD ± 7.4]) had a lower BMI (mean 24) and had more severe illness at initial presentation (average aggregate NEWS-2 score = 7, High clinic risk suggestive of urgent response with need for continuous monitoring) (Table 3.). Two of the deaths were complicated by the presence of sub-massive pulmonary emboli on admission, prior to initiating any therapy (Supplemental Table 1.).

**Table 3:** Baseline patient demographics and characteristics of COVID-19 positive inpatients treated with baricitinib/remdesivir/dexamethasone who did died during hospitalization. Plus–minus( ±) values are standard deviation

		Deceased Patients (n = 6)	
Age—years	Mean age (year)	84.5	SD ± 7.4
Gender—no	Male (n)	4	(66.7)
	Female (n)	2	(33.3)
Ethnicity – no. (%)	Not Hispanic, Latino/a, or Spanish origin (n)	6	(100.0)
Race – no. (%)	White (n)	5	(83.3)
	Asian (n)	0	-
	African American or Black (n)	1	(16.7)
	Multiracial (n)	0	-
	Declined (n)	0	-
Past Medical History—no. (%)	Co-existing conditions: None	0	-
	Co-existing conditions: One	0	-
	Co-existing conditions: More than 2	6	100.0
	Average BMI	24	
	Obesity: BMI: > 30	1	(16.7)
	Morbid Obesity: BMI: > 40	0	-
	Obstructive Sleep apnea	1	(16.7)
	Coronary artery disease	1	(16.7)
	Hypertension	4	(66.7)
	Type 2 Diabetes Mellitus	2	(33.3)
	Hyperlipidemia	2	(33.3)
	Heart failure (HFpEF, HFrEF or unspecified)	1	(16.7)
	Chronic Kidney Disease	1	(16.7)
	Atrial Fibrillation	3	(50.0)
	History of Malignancy	1	(16.7)
	Pulmonary hypertension	2	(33.3)
	Aortic stenosis	3	(50.0)
	Osteoarthritis	1	(16.7)
	Autoimmune disease (rheumatoid arthritis, psoriatic arthritis, Sjogren's Syndrome, Raynaud's phenomena, giant cell arteritis, ulcerative colitis, pemphigus vulgaris)	3	(50.0)
	Obstructive airway disease (asthma, chronic obstructive pulmonary disease, bronchiectasis and reactive airway disease)	2	(33.3)
Average duration of symptoms prior to presentation—days		3	(52.8)
Average severity of illness at presentation	NEWS-2 score	7	
	WHO Clinical Status Score	4	
Bilateral Pneumonia on imaging at presentation—no. (%)		4	(66.7)

Patient race and ethnicity are self-reported by patient. Body mass index is calculated by weight (kilograms) divided by square of height (meters). Percentages may not total 100 because of rounding

## Discussion

The current findings support the use of immunosuppressive therapy in moderate to severe hospitalized COVID-19 patients using a combination of corticosteroids, remdesivir, and a JAK1/2 inhibitor. All patients in this study also received remdesivir and dexamethasone in addition to baricitinib, so it is not possible to assess the individual contribution of each medication to recovery. This study spans a 6-month period during which SARS-CoV2 variants emerged in Vermont, USA.

Four patients required mechanical ventilation (8.9%) during their hospitalization. Of these, two received ventilatory support prior to starting baricitinib therapy, and one of these was extubated shortly after initiating baricitinib. This contrasts with the mean higher mechanical ventilation rate of 14.5% from a meta-analysis of 12,437 COVID-19 ICU admissions [28], and was considerably lower than our own experience in the early phases of the pandemic. The patient population in the current study was equally distributed between males and females, in contrast to many other studies. In addition, half were age 70 or older, which makes the favorable outcome all the more remarkable. Finally, there were six deaths (13.3%) and 11.1% 28-day mortality among the 45 patients. Two of these, however, were complicated by sub-massive pulmonary emboli present at the time of admission and prior to initiation of baricitinib. Our retrospective study did not have an untreated control group, but the mortality was lower than would be anticipated on review of the existing scientific literature. In a recent meta-analysis of 10,930 COVID-19 patients, the 28-day mortality risk was estimated at 25% for usual care or placebo [27].

Severe COVID-19 has close parallels with other seemingly unrelated syndromes that might collectively be classified as hyperinflammatory disorders [14]. Chimeric antigen receptor T (CAR-T) cell therapy exposes patients to a large number of T cells that become activated upon contact with targeted tumor cells, often resulting in a highly inflammatory cytokine release syndrome that can include hypercoagulation and even acute respiratory distress syndrome (ARDS) [29–31]. Toxic shock syndrome is a multi-organ inflammatory syndrome [32] in which tampons infected with *Staphylococcus* release an enterotoxin that acts as a superantigen by binding both the MHC class II molecule and the β-chain of several T cell receptors [33]. This activates a significant portion of the T cell repertoire, similar to CAR-T therapy, resulting in injury to many organs including skin, liver, and lung, and can also be associated with coagulopathy and ARDS [32]. Consistent with the view of hyperactivation of T cells in these disorders, individuals with HIV and low T cell counts have been noted to have less severe COVID-19 [34].

An additional parallel can be made between severe COVID-19 and hemophagocytic lymphohistiocytosis (HLH). HLH is a severe inflammatory syndrome characterized by fever, hepatitis, spleen and lymph node enlargement, and pancytopenia [35, 36]. It is often observed secondary to certain viral infections as well as autoimmune syndromes such as juvenile inflammatory arthritis [35]. An additional laboratory characteristic is elevated ferritin, which we observed in our severe COVID-19 cases. HLH is likely the result of strong T cell activation producing cytokines that activate macrophages to become highly phagocytic [35, 36]. Consequently, anti-cytokine therapy has also been used to treat HLH, including IL-1 blockade as well as JAK inhibitors.

This study sought to assess the efficacy and safety of 7 days of baricitinib treatment, whereas other recent trials in COVID-19 patients treated for 14 days [19–22]. Among our patients, the mean duration of baricitinib use was 6 days, and 37% of participants did not complete the full 7-day course as a consequence of discharge or clinical stabilization, suggesting that a 14-day course may not be required for improved clinical outcomes. The decision for a shorter treatment period was made out of a desire to balance the suppression of inflammation that might result in tissue damage with an avoidance of prolonged immunosuppression that might delay viral clearance or promote secondary infections. Previous work in rheumatoid arthritis patients receiving either biologic or targeted synthetic disease-modifying antirheumatic drugs (DMARDs) at the time of COVID-19 infection reported increased risk of hospitalization or death in patients using JAK inhibitors compared with either TNF inhibitors, IL-6 inhibitors or abatacept [37]. This highlights the dangers of prolonged immunosuppression, particularly at the outset of COVID-19 infection when type I interferon production is critical to control viral replication (Sparks et al., 2021). The appropriate timing of immunosuppression using JAK inhibition is thus critical, in order to preserve the early phase innate immune response, but inhibiting the late-phase cytokine storm response that can cause immune-mediated organ damage, at a time when viral titers are decreasing or even negligible. Additionally, delays in viral clearance have in fact been observed in immunocompromised patients, resulting in the emergence of viral variants [38]. Finally, given the known risk of JAK inhibitor-induced thrombosis, in the context of the recognized coagulopathy risk in COVID-19, a shorter treatment course may be favorable and sufficient for the duration of cytokine release syndrome in these patients.

Half of the patients with moderate to severe COVID-19 had a BMI greater than 30. This is considerably higher than the 23.2% obesity prevalence for the general population in Vermont (https://www.cdc.gov/obesity/stateprograms/fundedstates/pdf/vermont-state-profile.pdf). Obesity is a known risk factor for severe COVID-19 infection [39]. Obesity is also associated with a baseline inflammatory state [40]. Adipose tissue supports the development of tissue resident T lymphocytes that upregulate gene expression for several inflammatory cytokines as well as for cytolytic activity and express high levels of the checkpoint blocker programmed cell death protein-1 (PD-1) [41]. A very similar phenotype of T cells is observed in bronchiolar lavage fluid of COVID-19 patients [42]. Our retrospective review echoes prior work by the OpenSAFELY study that older age and medical comorbidities such as hypertension, obesity, pulmonary disease, and diabetes are associated with increased risk of hospitalization and poorer prognosis with COVID-19 infection [43].

No adverse effects were noted from the use of baricitinib. In particular, there were no secondary infections. Despite concern for increased thrombotic risk with baricitinib, we did not observe clinical evidence of new clots during the brief course of treatment, although two patients demonstrated significant clots on admission prior to initiation of baricitinib.

SARS-CoV-2 is known to suppress the initial IFN-I response, likely through the interaction of particular viral proteins with molecules of the IFN-I signaling pathway [44, 45]. This allows the virus to rapidly replicate during the early stages of infection. The delayed immune response can then become hyperactive and result in considerable cell death of surrounding tissues. This could include tissues that are not known to support SARS-CoV-2 replication, such as liver inflammation observed in some cases of severe COVID-19 [46]. The subsequent release of host RNA and DNA from damaged tissues can strongly activate, respectively, the retinoic acid-inducible gene 1 (RIG-I) and cyclic GMP-AMP synthase (cGAS) nucleic acid sensing pathways, leading to an augmented IFN-I response and persistent inflammation even in the absence of virus. This is consistent with studies showing that death of lung epithelium is due in some instances more to the immune response than to viral-mediated lysis [47]. Emerging evidence in animal models of SARS and MERS has revealed that the initial IFN-I response has beneficial effects in the early phases of disease but may become damaging in the latter phases [48].

In case series of patients with COVID-19, baricitinib treatment was associated with both an improvement in oxygenation and a reduction in select inflammatory markers [19–22]. The largest of these, the ACTT-2 Study Group, randomized 1033 patients to receive remdesivir and either baricitinib for up to 14 days (515) or placebo control (518). Patients receiving baricitinib had a median time to recovery of 7 days compared to 8 days for the control group and a 30% higher odds of improvement in clinical status at day 15. Patients receiving high-flow oxygen or non-invasive ventilation at enrollment had a time to recovery of 10 days with combination treatment and 18 days with control. The 28-day mortality was 5.1% in the combination group and 7.8% in the control group. These findings are echoed by a smaller randomized controlled trial reporting that a 14-day course of another JAK inhibitor, tofacitnib, was associated with reduced mortality and improved patient outcomes in patients hospitalized with COVID-19 pneumonia compared with placebo [18]. A recent meta-analysis of 5 randomized controlled trials using JAK inhibitors in hospitalized patients with COVID-19 similarly reported reduce mortality and improved clinical outcomes [49].

Limitations of the current study include its retrospective nature, a single-center study site, and the the lack of a untreated control group.

## Conclusions

We report a retrospective review of hospitalized COVID-19 patients treated with a short course of baricitinib in combination with dexamethasone and remdesivir. Consistent with previous literature, we report that an elevated BMI and multiple medical comorbidities are risk factors associated with hospitalization following COVID-19 infection. Additionally, older age, ≥ 2 co-existing medical conditions, and higher illness severity at time of presentation were associated with a poorer prognosis. Baricitinib use was associated with an increase in hemoglobin and platelet count, decrease in inflammatory markers (D-dimer, ferritin and CRP), and improvement in clinical status (specifically the WHO Clinical Status Score). The mean duration of baricitinib use was 6 days, which in light of the observed improvements in clinical status and inflammatory markers, may suggest that a shorter course could be beneficial while reducing the risk of more prolonged immunosuppression and thrombosis. No adverse effects were noted from the use of baricitinib. In particular, there were no secondary infections or thrombosis following initiation of baricitinib. The current findings support the use of immunosuppressive therapy in moderate to severe COVID-19 using a combination of steroids, remdesivir, and a JAK inhibitor.

## Supplementary Information

Below is the link to the electronic supplementary material.

Supplementary file1 (DOCX 22 KB)

## Data Availability

The datasets used and/or analyzed during the current study are available from the corresponding author on reasonable request.

## Abbreviations

AAK1: AP2-associated protein kinase
ARDS: Acute respiratory distress syndrome
ALT: Alanine transaminase
AST: Aspartate transaminase
CAR-T: Chimeric antigen receptor T
cGAS: Cyclic GMP-AMP synthase
COVID-19: Coronavirus Disease 2019
COPD: Chronic obstructive pulmonary disease
Cr: Creatinine
GFR: Glomerular filtration rate
GM-CSF: Granulocyte–macrophage colony-stimulating factor
HLH: Hemophagocytic lymphohistiocytosis
HFrEF: Heart failure reduced ejection fraction
HFpEF: Heart failure preserved ejection fraction
IFN-I: Type I Interferon
IL: Interleukin
IV: Intravenous
JAK: Janus kinase
MERS-CoV: Middle East respiratory syndrome coronavirus
NAK: Numb-associated kinase
NEWS-2: National Early Warning Score-2
PCR: Polymerase chain reaction
PD-1: Programmed cell death protein-1
RIG-I: Retinoic acid-inducible gene 1
SARS-CoV-1: Severe acute respiratory syndrome coronavirus 2
SARS-CoV-2: Severe acute respiratory syndrome coronavirus 2
SD: Standard deviation
WHO: World Health Organization
