# Supplementary material for: Efficacy of Combination Therapy with the JAK Inhibitor Baricitinib in the Treatment of COVID-19
Source: SN Compr Clin Med. 2022 Jan 21;4(1):42. doi: 10.1007/s42399-022-01121-4 (PMC8776555; doi:10.1007/s42399-022-01121-4)
Supplement: Supplementary file 1 — Supplementary file1 (DOCX 22 KB) [file 42399_2022_1121_MOESM1_ESM.docx]

| Supplementary Table 1. Patient demographics and details of treatment course of patients who died during hospitalization | | | | | | | | |
| --- | --- | --- | --- | --- | --- | --- | --- | --- |
| Gender | Age | BMI | Past Medical history | Details of COVID-19 therapy | Duration of Baricitinib treatment (days) | Duration of hospitalization (days) | Hospitalization course | Cause of death |
| Male | 85 | 23.9 | Coronary artery disease, heart failure with preserved ejection fraction, atrial fibrillation with sick sinus status post pacemaker, hypertension, Chronic kidney disease Stage 3, OSA on nocturnal BiPAP, intracranial hemorrhage status post VP shunt, seizures and cognitive decline | - Once daily Baracitinib 4mg x3, 2mg x2. - Once daily Dexamethasone 6mg x10. - Once daily Remdesivir 200mg x1, 100mg x4 | 5 | 47 | Multiple ICU admission secondary to respiratory decompensation attributed to mucous plugging and volume overload. Additionally developed parotitis and status epilepticus. | Acute hypoxic respiratory failure as a consequence of COVID-19 infection |
| Female | 86 | 17.5 | COPD, Raynaud's, atrial fibrillation, idiopathic peripheral neuropathy , CHF of unknown severity, prior breast Ca remission and osteoporosis | - Once daily Baricitinib 4mg x3 - Once daily Dexamethasone 6mg x3 - Once daily Remdisivir daily 200mg x1 100mg x2 | 3 | 6 | Acute hypoxic respiratory failure leading to transfer to mICU and intubation. Subsequent cardiac arrest. | Cardiac arrest as a consequence of COVID-19 infection |
| Male | 81 | 26.7 | COPD on 2L via nasal cannula at baseline, atrial fibrillation, type 2 diabetes mellitus, hyperlipidemia, giant cell arteritis, heart failure preserved ejection fraction and ulcerative colitis | - Once daily Baricitinib 4mg daily x5. - Once daily Dexametasone 6mg daily x6 - Once daily Remdesivir 200mg x1 and 100mg x2 | 5 | 10 | Hospital course complicated by bradycardia and hypotension. | Acute hypoxic respiratory failure secondary to pneumonia secondary COVID-19 |
| Female | 97 | 21.3 | Rheumatoid arthritis, osteoarthritis, hypertension, gastroesophageal reflux disease, depression and osteoarthritis (s/p bilateral hip arthroplasties) | - Once daily Baricitinib 4mg x1 2mg x6 - Once daily Dexamethasone 6mg x6. - Once daily Remdesivir 200mg x1 100mg x4 | 7 | 25 | Hospital course complicated by urosepsis and delirium following treatment with baricitnib. | Cardiac arrest as a consequence of hypovolemic shock as a consequence of COVID-19 infection |
| Male | 86 | 22.8 | Hypertension, hyperlipidemia, gastroesophageal reflux disease, osteoarthritis and benign prostatic hypertrophy | - Once daily Baricitinib 4mg x1. - Once daily Dexamethasone 6mg - Once daily Remdesivir 1x 200mg, 1x 100mg | 1 | 5 | Diagnosed with multiple subsegmental pulmonary emboli as well as multifocal pneumonia at time of presentation. CT angiogram identified a completely occlusive thrombus of the right common iliac, right profunda, and distal right common femoral extending into the popliteal artery. Surgical intervention was not consistent with patient's goals of care. | Cardiac arrest as a consequence of pneumonia as a consequence of COVID-19 infection |
| Male | 72 | 33.3 | Hypertension and Type 2 diabetes mellitus | - Patient received two 7-day courses of Baricitinib 4mg daily - Once daily Dexamethasone 6mg x14. - Once daily Remdesevir 200mg x1, 100mg x4 | 14 | 18 | Submassive pulmonary embolism at time of presentation and managed with enoxaparin. Treated for ventilator associated pneumonia. Course complicated by subglottic bleed requiring holding of anticoagulation. | Acute hypoxic respiratory failure secondary to acute respiratory distress syndrome secondary to COVID-19 pneumonia |

**Supplementary Table 1. Baseline patient demographics, details of hospital course and cause of death of COVID-19 positive inpatients treated with baricitinib / remdesivir / dexamethasone who died during hospitalization.** Body mass index is calculated by weight (kilograms) divided by square of height (meters). Cause of death as reported by pronouncing physician.

**Supplementary Figure 1. Baricitinib plus dexamethasone and remdesivir therapy decreases alanine transaminase levels and does not significant affect creatinine or aspartate transaminases levels.** Daily laboratory results were recorded from routine patient care including Creatinine (Cr) (g/dL) [A], Alanine transaminase (ALT) (U/L) [B] and Aspartate transaminase (U/L) [C]. The start date of baricitinib, dexamethasone and remdesivir therapy for each patient was arbitrarily set as day 0. All observations were divided into early (up to day -3) and late periods (up to day +9). Linear regressions were performed for each variable and a difference in slope tested between before treatment (day -3 to day 0) vs. after treatment (day +1 to day +9).
